# Chemical and Physicochemical Water Quality Parameters and Partial Least Squares Discriminant Analysis as Key Tools to Evaluate Dam Influence on Adjacent Surface Waters: Evidence from Bulgarian Reservoirs

**DOI:** 10.3390/molecules31101642

**Published:** 2026-05-13

**Authors:** Tony Venelinov, Galina Yotova, Aleksey Benderev, Stefan Tsakovski

**Affiliations:** 1 Faculty of Hydraulic Engineering, University of Architecture, Civil Engineering and Geodesy, 1046 Sofia, Bulgaria; tvenelinov_fhe@uacg.bg; 2 Faculty of Chemistry and Pharmacy, Sofia University “St. Kliment Ohridski”, 1164 Sofia, Bulgaria; g.yotova@chem.uni-sofia.bg; 3 Geological Institute, Bulgarian Academy of Sciences, 1113 Sofia, Bulgaria; alekseybenderev@yahoo.com

**Keywords:** dams, adjacent surface water bodies, water quality parameters, PLS-DA

## Abstract

Dam constructions alter the river flow, leading to a cascade of physical, chemical, and biological changes in the ecosystem’s structure and function. This study presents a systematic framework for assessing the impact of these built structures on adjacent surface water bodies. The approach integrates mandatory long-term monitoring data with a multivariate statistical approach (Partial Least Squares Discriminant Analysis, PLS-DA) to provide a robust assessment of fourteen of Bulgaria’s major and significant reservoirs’ influence on nearby rivers and streams. Datasets for studied reservoirs include basic physicochemical parameters, and for 8 out of 14 dams—potentially toxic elements (PTEs). To assess the influence of each reservoir on the river, two sampling locations were selected per dam: upstream (U) and downstream (D). Results for the water quality parameters, identified as significant discriminators in each PLS-DA model, are presented. A clear upstream dominance was observed for Pchelina, Saedinenie, and Ticha, a strong downstream pattern was observed for Dospat and Yovkovtsi, and a mixed spatial pattern for the remaining dams. The hierarchical clustering revealed three groups of parameters studied. The first cluster (EC, NO_2_^−^, NO_3_^−^, TN) likely reflects diffuse inputs. The second cluster (TP, PO_4_^3−^) describes the relationship between total and dissolved phosphorus fractions. The third cluster (pH, NH_4_^+^, DO, BOD) highlights organic matter decomposition and oxygen dynamics. The results highlight that reservoir impacts are governed by the interplay of hydrological conditions, catchment characteristics, and in-reservoir biogeochemical processes, leading to distinct functional behaviours such as retention, transformation, or release of substances.

## 1. Introduction

It is estimated that more than half of the world’s large rivers have already been influenced by damming, a figure anticipated to rise to 93% by 2030 [1]. Dams are constructed for various purposes—flood control, agricultural irrigation, domestic water supply, recreational activities and hydroelectricity generation [2]. As 71% of the global renewable energy supply is attributed to hydropower, it comes as no surprise that 3700 hydropower stations (exceeding 1 MW) are either planned or under construction worldwide [1]. The World Commission on Dams has concluded that the initial perception of dams as purely beneficial to society often overlooks their extensive ecological footprint—they transform natural riverine systems, leading to hydrological, morphological, and ecological impacts [2,3].

Rivers, in their natural state, are dynamic ecosystems characterised by continuous flow, seasonal sediment transport, and intricate nutrient cycling [4]. Dam constructions alter the river flow by changing it from lotic (flowing) to lentic (still), leading to a cascade of physical, chemical, and biological changes in the ecosystem’s structure and function [5]. 

They significantly alter the natural biogeochemical cycles of essential nutrients, such as carbon, nitrogen, silicon, and phosphorus. Sedimentation within the reservoir leads to increased nutrient retention, depriving downstream areas of the nutrient-rich sediment. This in-reservoir sedimentation was estimated to have wiped out 13% of the total riverine export of carbon to the oceans by the early 21st century, a value projected to rise to 19% by 2030 [3]. Water quality within impounded rivers deteriorates due to alterations in the dynamics of oxygen transfer mechanisms. Depletion in dissolved oxygen (DO) content is commonly observed, both in the vertical and longitudinal profiles of the river. Small dams, by slowing impoundment flow velocities, can reduce atmospheric mixing and turbulence, leading to reduced DO levels [6].

Other parameters, such as pH, nitrates, chlorides and electrical conductivity can also be affected, resulting in their reduction downstream of the reservoir [7]. Several studies documented increases in pH following dam construction. The Daechung Reservoir [8] showed pH increases from <7.5 to >7.5 across all reservoir zones after upstream dam construction. Similarly, pH increased in the Lancangjiang system [9], though the Nestos River showed slight pH decreases downstream [10].

Naeseong Stream exhibited high availability of nitrogenous compounds in the upstream dammed pool, with seasonal variations [11]. Nitrogen speciation also changed following dam construction. The increased hydraulic residence time in the Lancangjiang cascade system [9] and the anoxic conditions in the Nestos River [10] facilitated the transformation of dissolved inorganic nitrogen from nitrate-dominated to ammonium-dominated forms. Slower flow velocities caused by water impoundment promote particulate phosphorus deposition, leading to a significant decrease in total phosphorus (TP) concentrations after dam construction. Sharp phosphorus declines downstream were observed in the Yeongsan River [12], the Lancangjiang system [9], the Geum River [13] and Yangtze River [14], where the dams acted as a sink for phosphorus.

The presence and dynamics of trace and potentially toxic elements (PTEs) constitute a critical environmental concern in dammed river systems, given their persistence, toxicity, and capacity for bioaccumulation [15]. These elements originate from both natural geological processes (e.g., mineral weathering, volcanic activity, erosion of parent rocks) and anthropogenic activities (mining, metallurgy, industrial discharges, the use of pesticides and fertilisers in agriculture, sewage sludge disposal, vehicle exhaust emissions, and rapid urbanisation), leading to elevated concentrations in aquatic environments [16]. PTEs in river systems possess a strong affinity for particulate matter. Only a small fraction of them remains in the dissolved state within the water column; the vast majority adsorbs onto particles and enriches the sediment, resulting in significantly higher concentrations in sediment than in the overlying water. Sediments thus act as major, long-term sinks for PTEs due to their low solubility in water and their easy absorption onto fine particles [17]. Changes in hydrodynamic conditions (e.g., increased flow velocity, turbulence from dam operations) or alterations in key environmental factors (such as pH [18], redox potential [17] and organic matter concentration [19]) can lead to the re-release and remobilisation of PTEs from the sediment back into the water column, causing significant secondary pollution [20,21]. PTE concentrations in river sediments can increase from upstream to downstream. For instance, in the Three Gorges Reservoir, most PTEs (except Ni and As) showed an increase with Cd concentrations, approximately doubling compared to pre-impoundment levels [17].

Bulgarian dams were studied over the years [22,23,24,25,26,27,28,29,30,31,32,33,34,35,36], mainly for their water quality, rather than for their influence on the adjacent surface water bodies. Several surface water sampling campaigns for monitoring of rivers have been carried out, but without analysing the impact of the constructed dams.

However, to our knowledge, no study has yet established a systematic framework for assessing the impact of these built structures on adjacent surface water bodies. The aim of this study is, therefore, to develop and demonstrate such a framework. We propose a method that integrates mandatory long-term monitoring data in compliance with the EU Water Framework Directive (WFD) with a multivariate statistical approach—specifically, Partial Least Squares Discriminant Analysis (PLS-DA)—to provide a robust assessment of dam influence on nearby rivers and streams.

## 2. Results

### 2.1. Input Data Arrangement

**Figure 1:**
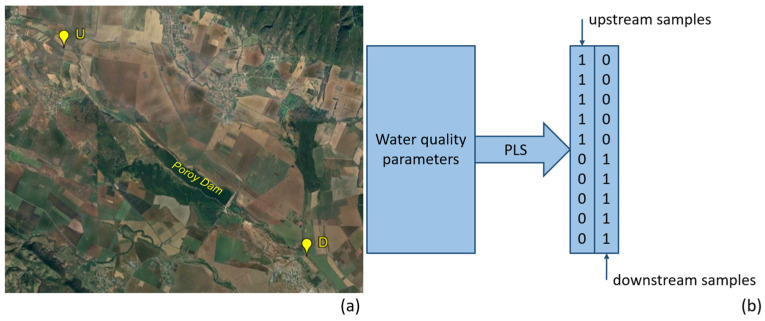
Sampling and analyses strategy: (**a**) Sampling map for Poroy Dam: U—upstream sample; D—downstream sample; (**b**) Illustration for PLS-DA analysis for modelling including two classes.

Fourteen of Bulgaria’s major and significant reservoirs were examined in this study. To assess the influence of each reservoir on the river, two sampling locations were selected per dam: one upstream (U) and one downstream (D). Figure 1a illustrates the sampling design for the Poroy Dam as an example.

For each of the studied dams, datasets were compiled containing water quality measurements from the upstream and downstream locations collected over multiple years between 2010 and 2024. These datasets served as the input for PLS-DA classification models, which were constructed to distinguish between upstream and downstream samples for each reservoir (Figure 1b). The resulting models enabled the identification of patterns in water quality changes associated with the reservoir.

### 2.2. PLS-DA Models

**Figure 2:**
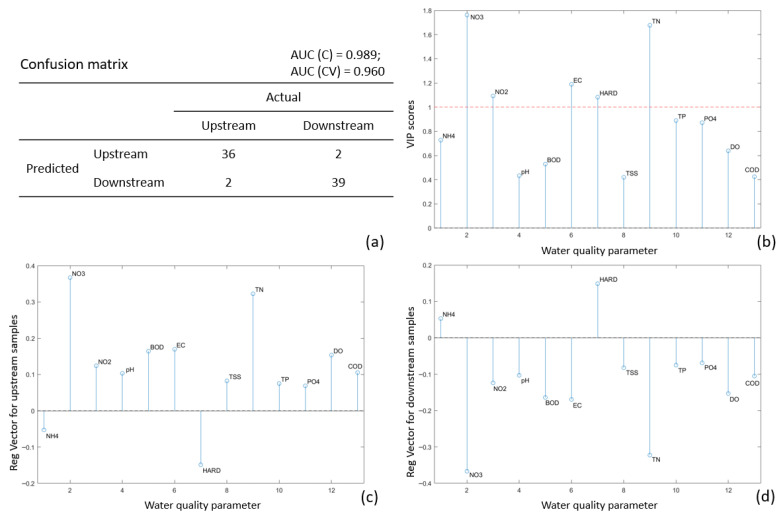
The partial least squares-discriminant analyses model results for Poroy Dam: (**a**) Confusion matrix; (**b**) VIP (variable importance on projection) scores; (**c**) Regression vector for upstream samples; (**d**) Regression vector for downstream samples.

The PLS-DA classification results for the Poroy Dam are presented in Figure 2 as an illustrative example. The confusion matrix indicates that only four samples (5.06%) were misclassified (Figure 2a), demonstrating high model accuracy. The area under the curve (AUC) values of 0.989 (C—calibration) and 0.960 (CV—cross-validation) indicate excellent prediction performance. Water quality parameters with a significant contribution to the classification model are identified by a Variable Importance in Projection (VIP) value greater than 1. For the Poroy Dam, five of the thirteen parameters were highly influential: nitrates (NO_3_^−^), nitrites (NO_2_^−^), electrical conductivity (EC), hardness (HARD), and total nitrogen (TN) (Figure 2b). Parameters with VIP values above 0.8, including total phosphorus (TP) and phosphates (PO_4_^3−^), also exhibit a supporting influence in the classification. The regression vectors for upstream (Figure 2c) and downstream (Figure 2d) samples represent the concentration profiles of each class. Considering the significant parameters, upstream samples exhibit higher levels of NO_3_^−^, NO_2_^−^, EC, TN, TP and PO_4_^3−^, whereas downstream samples show higher hardness (HARD).

The discriminant performance of the PLS-DA models, as evaluated by the area under the curve (AUC), varied across the studied reservoirs (Table 1). The model for Topolnitsa Dam achieved perfect classification (AUC = 1.0). Most other models demonstrated excellent performance (AUC > 0.9). Cross-validation results indicated good predictive ability (0.8 < AUC < 0.9) for Ticha and Tsonevo, fair ability (0.7 < AUC < 0.8) for Yovkovtsi, and poor ability (0.6 < AUC < 0.7) for Ogosta.

The input datasets for each reservoir consisted of 11 to 21 water quality parameters, which included a core set of 10 physicochemical variables (pH, EC, DO, NH_4_^+^, NO_3_^−^, NO_2_^−^, TN, TP, PO_4_^3−^, BOD). For many of the classification models, VIP scores identified more than 50% of the analysed water quality parameters as significant contributors (VIP > 0.8) to group separation. Notable exceptions to this pattern were observed in the Ogosta, Saedinenie, Studen Kladenets, and Yovkovtsi reservoirs, where a substantially smaller subset of variables was identified as significant.

Table 2 presents the water quality parameters identified as significant discriminators in each PLS-DA model. The direction of influence (U for higher upstream, D for higher downstream) is derived from the regression vector coefficients, while colour shading denotes the level of significance.

The analysis of physicochemical parameters revealed distinct patterns. Dissolved oxygen (DO) was consistently elevated in downstream samples across all models, where it was a significant discriminator. As anticipated, total phosphorus (TP) and phosphates (PO_4_^3−^) showed congruent behaviour, consistently exhibiting the same directional influence (i.e., both higher upstream or both higher downstream) in every model. Furthermore, potentially toxic elements (PTEs) were significant discriminators in six of the eight dam datasets where they were measured. While most PTEs were predominantly elevated upstream, notable exceptions were observed for arsenic (As), iron (Fe), manganese (Mn), and nickel (Ni), which demonstrated higher downstream concentrations in several instances.

A dam-specific analysis further elucidated distinct spatial patterns in parameter influence. A clear upstream dominance was observed for three reservoirs (Pchelina, Saedinenie, and Ticha), where all significant parameters were elevated. Conversely, the reservoirs at Dospat and Yovkovtsi exhibited a strong downstream pattern, with all key discriminators showing higher concentrations. For the remaining dams, the significant parameters reflected a mixed spatial pattern, with both upstream and downstream dominance.

**Figure 3:**
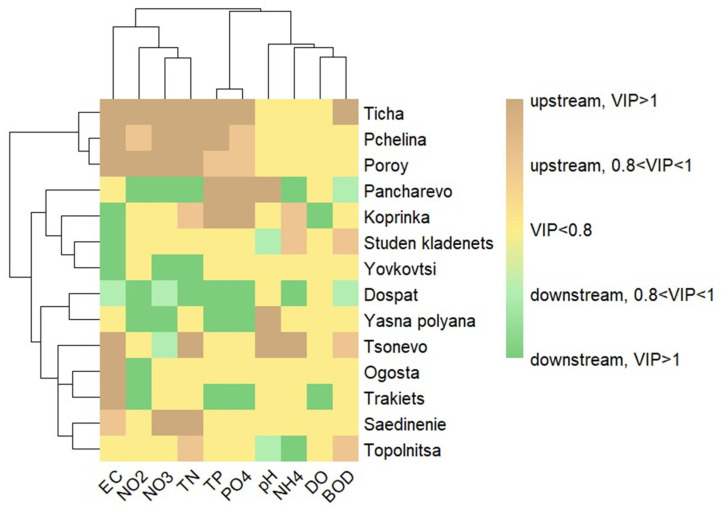
Clustered heatmap of VIP scores of 10 physicochemical parameters for 14 dams.

To identify patterns among the studied dams, a clustered heatmap was generated based on their water quality profiles (Figure 3). The analysis incorporated 10 physicochemical parameters common to all 14 reservoirs. The input data were derived from the PLS-DA models, where each parameter was assigned a score reflecting its significance and directional influence: parameters with a VIP score >1 were coded as +1 (upstream-prevailing) or −1 (downstream-prevailing); those with 0.8 < VIP < 1 were coded as +0.5 or −0.5, respectively; and non-significant parameters (VIP < 0.8) were coded as 0. Hierarchical clustering of both dams and parameters, using Euclidean distance and Ward’s linkage method, revealed distinct clusters of reservoirs with similar discriminatory patterns.

Hierarchical clustering based on the signed VIP scores revealed four distinct clusters of dams, reflecting shared patterns in the significance and upstream/downstream prevalence of physicochemical parameters (Figure 3). Cluster 1 (Ticha, Pchelina, Poroy) was characterised by a consistent profile where a suite of parameters—EC, TP, PO_4_^3−^, NO_2_^−^, NO_3_^−^, and TN—were significant and exhibited higher upstream concentrations. In contrast, Cluster 3 (Dospat, Yasna Polyana) displayed an inverse pattern, with the most significant parameters prevailing downstream. Cluster 2 (Pancharevo, Koprinka, Studen Kladenets, Yovkovtsi) showed no single dominant pattern, with primary similarities observed between pairwise combinations. Cluster 4 (Tsonevo, Ogosta, Trakiets, Saedinenie, Topolnitsa) presented a mixed signature, sharing some upstream trends with Cluster 1 but with considerable inconsistency. The clustering of Saedinenie within this group, despite its exclusive upstream profile, underscores that the grouping was driven by overall profile similarity (a small number of significant parameters) rather than any single characteristic.

The hierarchical clustering of parameters delineated three distinct groups. The first cluster (EC, NO_2_^−^, NO_3_^−^, TN) combines ionic strength with nitrogen species, indicating a shared pattern likely reflecting diffuse inputs and catchment-scale processes influencing both salinity and nitrogen transport. The second cluster (TP, PO_4_^3−^) forms a tightly coupled pair, demonstrating strong co-variation and consistent behaviour, which is expected given the direct relationship between total and dissolved phosphorus fractions. The third cluster (pH, NH_4_^+^, DO, BOD) groups parameters associated with in situ biogeochemical processes, particularly organic matter decomposition and oxygen dynamics, suggesting a common control by internal reservoir conditions rather than external loading.

## 3. Discussion

In contrast to univariate statistical approaches [8,12,14,37], which assess individual water quality indicators independently and do not account for inter-parameter interactions, PLS-DA provides a multivariate framework that simultaneously evaluates all variables to identify the optimal combination discriminating predefined groups (upstream vs. downstream). Univariate methods are further limited by the multiple testing problem and offer limited insight into the combined behaviour of indicators. In contrast, PLS-DA effectively handles collinearity, reduces data dimensionality, and enables the identification of the most influential variables through variable importance in projection (VIP) scores, thereby providing a more integrative interpretation of the processes governing water quality changes across reservoir systems.

Electrical conductivity (EC) was the most significant discriminator, with a VIP score > 0.8 in 11 of the 14 models. As a composite measure of total dissolved ions, upstream/downstream differences in EC can be attributed to a multitude of factors, reflecting the complex influence of the reservoirs on water quality. A significant downstream decrease in EC was observed for 7 of the 11 dams. The most prevalent mechanism for this reduction is likely reservoir stratification, where water released from the oxygenated, lower-TDS (total dissolved solids) epilimnion leads to a freshening effect downstream [7]. This pattern was particularly evident in larger reservoirs (e.g., Ticha, Tsonevo, Ogosta) or those with a stagnant regime fed by smaller rivers (e.g., Trakiets, Poroy, Pchelina, Saedinenie).

Additional catchment-specific factors further contributed to the observed decrease in EC. In smaller reservoirs, such as Saedinenie and Poroy, the underlying geology—characterised by weathering to clay-rich materials—likely acts as a sink for dissolved ions through adsorption onto fine sediment particles. Furthermore, the inflow of low-mineralisation water from mountain tributaries exerts a significant dilution effect in the catchments of reservoirs such as Ogosta and Ticha. The observed downstream increase in EC at the Koprinka, Studen Kladenets, Dospat, and Yovkovci reservoirs is likely attributable to the internal reservoir processes such as evaporative concentration and the dissolution of minerals. Furthermore, some of the systems (Dospat and Yovkovtsi) are susceptible to point and non-point source pollution, which contributes an additional ionic load to the reservoir and subsequently elevates the EC of the released water.

The mechanisms responsible for significant EC shifts in the eleven dams provide a framework for understanding similar variations in other water quality parameters (Table 2), except for PTEs. Upstream dominance in a parameter is generally attributable to reservoir stratification and sediment trapping. Conversely, downstream dominance often results from internal biogeochemical processes within the reservoir, and, in the case of nutrients and total organic carbon (TOC), pollution sources in the catchment. A notable case is the downstream increase in DO, which is a direct result of stratification that allows for the release of oxygen-rich water from the reservoir’s surface layer.

Total organic carbon emerged as the most significant discriminating variable in all PLS-DA dam models with sufficient data (4 out of 14). This primacy, coupled with TOC’s fundamental role in modulating the behaviour and bioavailability of other pollutants, underscores its pivotal value. The relevance of TOC is further amplified by its strong correlation with dissolved organic carbon (DOC), the fraction that directly influences metal speciation [38]. This allows TOC to serve as a robust operational surrogate for DOC in Biotic Ligand Models (BLMs), which are critical for predicting the bioavailability and toxicity of PTEs. Consequently, TOC monitoring is indispensable for a practical and robust environmental risk assessment.

The environmental changes caused by dam construction on rivers facilitate the accumulation of PTEs in surface reservoir sediments [39], which explains their increasing concentration upstream. The fate of trapped PTEs in reservoir sediments is governed by multiple factors and may result in their remobilization into the water column, leading to elevated concentrations in downstream releases. The downstream increase of Fe and As in Topolnitsa Dam and of As and Ni in Trakiets Reservoir is likely driven by a combination of anoxic conditions within the reservoirs and the persistent influx of contaminants from ore deposits areas in their catchments. Reliable risk assessment in reservoir-containing river basins requires monitoring Fe and Mn, as the reductive dissolution of their oxides is a primary mechanism driving the remobilisation of co-precipitated PTEs from sediments.

The heatmap-based clustering of dams confirms that spatial patterns in water quality are not governed by single parameters but by integrated system behaviour reflecting the interplay between hydrological regime, catchment characteristics, and in-reservoir processes. The separation into upstream-dominated, downstream-dominated, and mixed-response clusters indicates that reservoir’s function either as sinks, transformers, or sources of key physicochemical parameters, depending on local conditions. Systems exhibiting consistent upstream dominance are indicative of efficient sedimentation and stratification-driven retention, whereas downstream-dominated reservoirs reflect stronger internal processing and/or continued external pollution inputs. The mixed clusters further emphasize that transitional or system-specific controls can override generalized behaviour. Importantly, the clustering demonstrates that reservoirs with similar sizes or geographic settings do not necessarily exhibit similar functional responses, underscoring the need for site-specific assessment.

## 4. Materials and Methods

### 4.1. Data Acquisition

Water resource management in Bulgaria is regulated by the Water Act [40], under which a list of 52 complex and significant dams has been established (last revised 2010). Monitoring data from national control programmes were compiled for the period 2010–2024. To assess dam impacts on adjacent rivers, two sampling points were selected for each reservoir: one upstream and one downstream.

Surface water monitoring follows Regulation No. 1 of 11.04.2011 [41], which specifies type-specific reference conditions and classification systems for ecological status assessment. Site selection was based on: (i) adequacy of monitoring periods and available parameters, (ii) proximity to the reservoir, and (iii) absence of significant anthropogenic pressures between the dam and sampling location.

**Figure 4:**
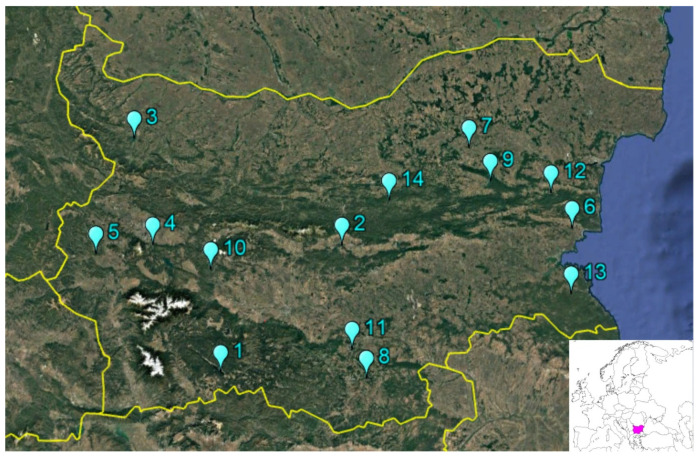
Sampling locations of the dams.

Applying these criteria, 14 reservoirs were included in the present study (Figure 4). The sampling period, storage capacity, and water use of the reservoirs are summarised in Table 3.

### 4.2. Water Quality Parameters

**Figure 5:**
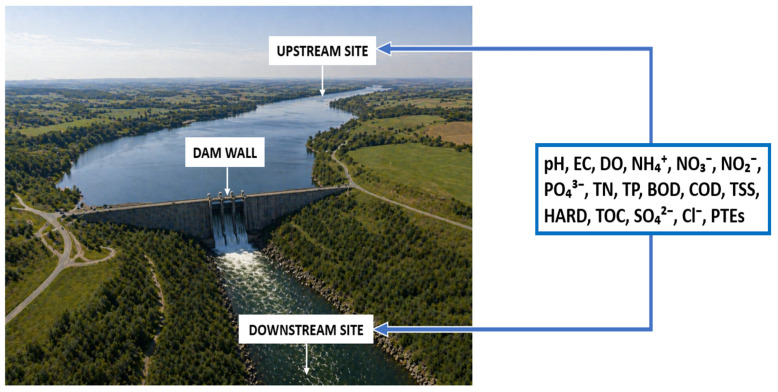
Sampling scheme for all the dams.

Mandatory surface water monitoring in Bulgaria is conducted in compliance with the EU Water Framework Directive [42] and national legislation, including the Water Act and associated regulations. Control monitoring at each sampling point encompasses biological, hydromorphological, and basic physicochemical quality parameters. In addition, depending on site-specific conditions, analyses include priority substances and other pollutants discharged in significant quantities within the relevant river basin (Figure 5).

For all studied reservoirs, the compiled dataset includes the following basic physicochemical parameters: pH, electrical conductivity (EC), dissolved oxygen (DO), NH_4_^+^, NO_3_^−^, NO_2_^−^, PO_4_^3−^, total nitrogen (TN), total phosphorus (TP), and biochemical oxygen demand (BOD). Most sampling sites also report chemical oxygen demand (COD), total suspended solids (TSS), and hardness (HARD), while some provide additional data for total organic carbon (TOC), SO_4_^2−^, and Cl^−^. Furthermore, datasets for eight reservoirs include concentrations of potentially toxic elements (PTEs), specifically Al, As, Cd, Cu, Fe, Mn, Ni, Pb, and Zn (Table 4).

All analyses were performed in accredited laboratories in accordance with Bulgarian national regulations and established quality standards [41,43].

### 4.3. PLS-DA Analysis

Partial least squares-discriminant analysis (PLS-DA) is a widely recognized classification and pattern recognition technique in chemometrics [44,45], although its application in environmental assessment studies is still limited [46,47]. PLS-DA is a specific variant of partial least squares modelling that combines the extraction of PLS components (latent variables, LVs) with discriminant analysis in the reduced PLS space.

PLS-DA is used to optimise the separation between different groups by linking the matrix of independent variables X (water quality parameters) with the corresponding matrix of dependent categorical variables Y (class membership, e.g., upstream and downstream samples). Prior to analysis, all input data were autoscaled, and cross-validation was performed using the Venetian blinds method.

The main objective of PLS-DA is to build a model that distinguishes between sample groups and identifies which variables are most important for classification. The importance of each independent variable in the prediction model is assessed using the Variable Importance in Projection (VIP) score. Water quality parameters with VIP values greater than 1 are considered highly influential in the classification model, while variables with VIP values above 0.8 can also be interpreted as significant [48]. The resulting regression vectors describe the variable profiles of the known classes (upstream vs. downstream samples).

Model performance was primarily evaluated using the area under the receiver operating characteristic (ROC) curve (AUC). The ROC curve combines classification performance metrics by plotting the sensitivity (true positive rate) against 1-specificity (false positive rate). Two parameters are typically reported: AUC(C) and AUC(CV). AUC(C) is calculated from predictions obtained using the full calibration dataset, where the same samples are used both to construct the model and to evaluate the performance. The predicted class membership scores are used to generate the ROC curve, from which the AUC is computed. AUC(C) reflects the model’s goodness of fit but often leading to optimistic estimates due to potential overfitting. In contrast, AUC(CV) is derived from cross-validation, where each sample is predicted by a model that excludes it from the training step, providing a more reliable estimate of predictive performance.

Predictive performance can be categorised as “excellent” (AUC > 0.9), “good” (0.9 > AUC > 0.8), “fair/acceptable” (0.8 > AUC > 0.7), “poor” (0.7 > AUC > 0.6), and “fail” (0.6 > AUC > 0.5) [49].

All multivariate statistics models were performed in MATLAB R2021a using PLS Toolbox 9.0 (Eigenvector Research Inc., Manson, WA, USA).

### 4.4. Cluster Analysis

To visualize the class-specific importance of physicochemical parameters across the sampled dams, a heatmap was generated using the pheatmap package (v1.0.12) in R. The input matrix consisted of rows representing individual dams and columns representing physicochemical parameters. Each cell value was derived from the VIP scores of a preceding PLS-DA model, which classified samples as ‘upstream’ or ‘downstream’. Following standard practice, these VIP scores were signed to indicate the class in which each parameter’s concentration was predominantly higher; positive values were assigned to parameters more abundant upstream, and negative values to those more abundant downstream. Unsupervised hierarchical clustering was performed on both rows (dams) and columns (parameters) using Euclidean distance and Ward’s linkage method (method = “ward.D2”). The diverging colour gradient, from green to brown, represents this signed VIP information, where the hue indicates the class association (upstream vs. downstream) and the intensity corresponds to the magnitude of the VIP score.

## 5. Conclusions

The present study demonstrates that the integration of Partial Least Squares Discriminant Analysis (PLS-DA) with routinely collected monitoring data, compliant with the EU Water Framework Directive (WFD), provides a robust and effective framework for assessing the influence of dams on adjacent surface water bodies. The application of signed VIP scores enabled the identification of key discriminating parameters and their spatial patterns, providing detailed insight into upstream and downstream processes across diverse reservoir systems.

The results highlight that reservoir impacts are governed by the interplay of hydrological conditions, catchment characteristics, and in-reservoir biogeochemical processes, leading to distinct functional behaviours such as retention, transformation, or release of substances.

From a water management perspective, the proposed framework provides actionable insights for optimizing monitoring strategies through the targeted inclusion of key parameters such as TOC, Fe, Mn, and site-specific pollutants, thereby enabling more precise and context-dependent assessments. Furthermore, the identification of dominant spatial patterns supports improved dam operation practices, particularly regarding the regulation of water release (e.g., withdrawal depth and timing), and informs the design of measures aimed at maintaining or achieving good chemical and ecological status of reservoirs and downstream water bodies.

## Figures and Tables

**Table 1:** Summary of the PLS-DA model results for 14 dams.

No.	Dam	Samples	AUC (C)	AUC (CV)	Parameters	VIP > 1	0.8 > VIP > 1	LVs
1	Dospat	59	0.998	0.980	11	5	3	1
2	Koprinka	35	1.000	0.917	11	4	2	4
3	Ogosta	84	0.766	0.698	17	5	0	1
4	Pancharevo	34	0.969	0.906	20	7	4	2
5	Pchelina	112	0.998	0.997	11	5	2	2
6	Poroy	79	0.989	0.960	13	5	2	3
7	Saedinenie	56	0.982	0.967	14	4	2	1
8	Studen Kladenets	29	0.991	0.914	16	3	3	1
9	Ticha	51	0.922	0.840	12	7	2	1
10	Topolnitsa	27	1.000	1.000	21	9	4	1
11	Trakiets	22	1.000	0.962	20	10	1	4
12	Tsonevo	41	0.991	0.895	18	7	4	2
13	Yasna Polyana	64	0.993	0.945	18	9	0	1
14	Yovkovtsi	51	0.928	0.797	17	7	1	1

**Table 2:** Summary of significant parameters and their directional influence (U/D) from dam-specific PLS-DA models.

No.	1	2	3	4	5	6	7	8	9	10	11	12	13	14
Dam/ Water Quality Parameter	Dospat	Koprinka	Ogosta	Pancharevo	Pchelina	Poroy	Saedinenie	Studen Kladenets	Ticha	Topolnitsa	Trakiets	Tsonevo	Yasna Polyana	Yovkovtsi
pH *				U				*D*		*D*		U	U	
**EC**	*D*	D	U		U	U	*U*	D	U		U	U		D
**DO**		D									D			
**NH_4_^+^**	D	*U*		D				*U*		D		U		
**NO_3_^−^**	*D*			D	U	U	U		U			*D*	D	D
**NO_2_^−^**	D		D	D	*U*	U			U		D		D	
**TN**	D	*U*		D	U	U	U		U	*U*		U		D
**TP**	D	U		U	U	*U*			U		D		D	
**PO_4_^3−^**	D	U		U	*U*	*U*			U		D		D	
**BOD**	*D*			*D*				*U*	U	*U*		*U*		
**COD**										U		U	D	
**TSS**					U		U		*U*					*D*
**HARD**			U			D	U	D	*U*		D	D	D	D
**TOC**				*U*			*U*					*U*	D	
**SO_4_^2−^**			U									U		D
**Cl^−^**				*U*								*U*	D	D
**Al**										U				
**As**										D	D			
**Cd**											U			
**Cu**										U				
**Fe**				*U*						*D*	*U*			
**Mn**			D					U		U				D
**Ni**										U	D			
**Pb**										U				
**Zn**										U	U			

* The direction of influence (U: upstream, D: downstream) is indicated by colour (brown and green, respectively). The significance level, based on the Variable Importance in Projection (VIP) score, is represented by colour intensity and italics: solid (VIP > 1.0), light/italicized (0.8 < VIP < 1.0). Non-significant parameters (VIP < 0.8) are shaded yellow.

**Table 3:** Characteristics of the studied reservoirs: monitoring period, sample size, storage capacity, and primary water uses.

No.	Dam	Period	No. of Samples	Total Storage Capacity in Billion m^3^	Use			
					Water Supply	Energy Production	Irrigation	Other *
1	Dospat	2013–2022	59	449.2				+
2	Koprinka	2018–2023	35	142.2	+	+	+	
3	Ogosta	2010–2021	84	506.0	+	+		
4	Pancharevo	2016–2024	34	6.5	+	+		
5	Pchelina	2010–2024	112	54.2				+
6	Poroy	2013–2024	79	45.2				+
7	Saedinenie	2013–2022	56	12.8				+
8	Studen Kladenets	2018–2022	29	387.8		+		
9	Ticha	2017–2023	51	311.8	+	+		
10	Topolnitsa	2016–2021	27	137.1				+
11	Trakiets	2016–2019	22	114.0				+
12	Tsonevo	2013–2022	41	330.0	+	+		
13	Yasna Polyana	2013–2021	64	32.3	+			
14	Yovkovtsi	2010–2016	51	92.2	+	+		

* Other—aquaculture and fishing; swimming and water sports; tourism, camping and recreational activities.

**Table 4:** Water quality parameters included in the data analysis for each dam.

No.	1	2	3	4	5	6	7	8	9	10	11	12	13	14
Dam/ Water Quality Parameter	Dospat	Koprinka	Ogosta	Pancharevo	Pchelina	Poroy	Saedinenie	Studen Kladenets	Ticha	Topolnitsa	Trakiets	Tsonevo	Yasna Polyana	Yovkovtsi
**pH**	+	+	+	+	+	+	+	+	+	+	+	+	+	+
**EC**	+	+	+	+	+	+	+	+	+	+	+	+	+	+
**DO**	+	+	+	+	+	+	+	+	+	+	+	+	+	+
**NH_4_^+^**	+	+	+	+	+	+	+	+	+	+	+	+	+	+
**NO_3_^−^**	+	+	+	+	+	+	+	+	+	+	+	+	+	+
**NO_2_^−^**	+	+	+	+	+	+	+	+	+	+	+	+	+	+
**TN**	+	+	+	+	+	+	+	+	+	+	+	+	+	+
**TP**	+	+	+	+	+	+	+	+	+	+	+	+	+	+
**PO_4_^3−^**	+	+	+	+	+	+	+	+	+	+	+	+	+	+
**BOD**	+	+	+	+	+	+	+	+	+	+	+	+	+	+
**COD**		+	+	+		+	+	+		+	+	+	+	+
**TSS**	+		+	+	+	+	+		+			+	+	+
**HARD**			+	+		+	+	+	+	+	+	+	+	+
**TOC**				+			+					+	+	
**SO_4_^2−^**			+	+								+	+	+
**Cl^−^**			+	+								+	+	+
**Al**				+						+				
**As**										+	+			
**Cd**										+	+			
**Cu**				+				+		+	+			
**Fe**			+	+				+		+	+	+	+	+
**Mn**			+	+				+		+	+	+	+	+
**Ni**										+	+			
**Pb**										+	+			
**Zn**								+		+	+			

## Data Availability

The raw data were derived from the Open Data Portal (data.egov.bg): Bulgarian public data in open and machine-readable format [https://data.egov.bg/organisation/e3d162cf-10ab-4808-a065-cd5380fbafca/datasets?q=%D0%BC%D0%BE%D0%BD%D0%B8%D1%82%D0%BE%D1%80%D0%B8%D0%BD%D0%B3&page=2, accessed on 10 May 2026].

## Abbreviations

The following abbreviations are used in this manuscript:

WFD	Water Framework Directive
PLS-DA	Partial least squares-discriminant analysis
AUC	Area under the curve
C	Calibration
CV	Cross-validation
VIP	Variable Importance in Projection
U	Upstream
D	Downstream
PAH	Polycyclic aromatic hydrocarbons
EC	Electrical conductivity
DO	Dissolved oxygen
TN	Total nitrogen
TP	Total phosphorus
BOD	Biochemical oxygen demand
COD	Chemical oxygen demand
TSS	Total suspended solids
HARD	Hardness
TOC	Total organic carbon
DOC	Dissolved Organic Carbon
TDS	Total dissolved solids
PTEs	Potentially toxic elements
